# Sewing Machines

**Published:** 1870-11

**Authors:** 


### SEWING MACHINES.

There is nothing in the working of a sewing machine calculated to impair the health; the labor is uniform, not excessive, nor calculated to overstrain any of the muscles of the system. A contrary view was taken at one time, but it was formed from incomplete observations, and a want of care in ascertaining all the facts in given cases. The motion of the feet promotes the circulation, and tends to keep them warm; the motion of the fingers invites the blood to the extremities, which all admit is a healthful observation, while the necessity of adjusting the work keeps the mind on the alert.

